# Correlation of objective image quality and working length measurements in different CBCT machines: An ex vivo study

**DOI:** 10.1038/s41598-020-76424-4

**Published:** 2020-11-10

**Authors:** T. G. Wolf, F. Fischer, R. K. W. Schulze

**Affiliations:** 1 Department of Restorative, Preventive and Pediatric Dentistry, School of Dental Medicine, University of Bern, Bern, Switzerland; 2 Department of Periodontology and Operative Dentistry, University Medical Center of the Johannes Gutenberg-University Mainz, Mainz, Germany; 3 Private Clinic for Oral and Maxillofacial Surgery, Koblenz, Germany; 4 Department of Oral and Maxillofacial Surgery, University Medical Center of the Johannes Gutenberg-University Mainz, Mainz, Germany

**Keywords:** Quality control, Oral anatomy, Applied physics, Imaging techniques

## Abstract

To investigate potential correlations between objective CBCT image parameters and accuracy in endodontic working length determination ex vivo. Contrast-to-noise ratio (CNR) and spatial resolution (SR) as fundamental objective image parameters were examined using specific phantoms in seven different CBCT machines. Seven experienced observers were instructed and calibrated. The order of the CBCTs was randomized for each observer and observation. To assess intra-operator reproducibility, the procedure was repeated within six weeks with a randomized order of CBCT images. Multivariate analysis (MANOVA) did not reveal any influence of the combined image quality factors CNR and SR on measurement accuracy. Inter-operator reproducibility as assessed between the two observations was poor, with a mean intra-class correlation (ICC) of 0.48 (95%-CI  0.38, 0.59) for observation No. 1. and 0.40 (95%-CI 0.30, 0.51) for observation No. 2. Intra-operator reproducibility pooled over all observers between both observations was only moderate, with a mean ICC of 0.58 (95%-CI 0.52 to 0.64). Within the limitations of the study, objective image quality measures and exposure parameters seem not to have a significant influence on accuracy in determining endodontic root canal lengths in CBCT scans. The main factor of variance is the observer.

The success of endodontic treatment, among other factors, is strongly correlated to accurate determination of the endodontic working length^1^. While the position of the physiological foramen during root canal treatment cannot be determined with absolute certainty, a combination of different methods could enhance accuracy^2^. Anatomical differences in physiological foramen geometry must be cautiously considered when making a final decision on clinical endodontic treatment^3^ to avoid over-instrumentation or insufficient root canal treatment^4^. Electronic apex locators offer precise results for working length^5^ and are considered a reliable method that can reduce the number of radiographs required^6^. A combination of electronic and radiographic methods is commonly used; however, cone-beam computed tomography (CBCT) images providing additional information often exist for patients prior to an endodontic treatment^7–9^. It has been proposed that endodontic working length determination can be done rather accurately by means of CBCT compared to electronic apex locators^7,8,10^. Yet, the variance between different CBCT devices is well known^11,12^. More precisely, hardware, exposure parameters, field of view size and reconstruction parameters differ to a great extent between different machines, and thus no two CBCTs are the same^12^. These different characteristics affect image quality, dose ranges^13^ and probably the interpretation of the images as well. The latter is the reason why quality control standards or CBCT have been set up^14^ or suggested by expert groups^15^. On the other hand, it is not an effortless task to establish a direct link between objective image quality and a specific diagnostic task. Objective image parameters for CBCT comprise contrast, spatial resolution (SR), noise characteristics and homogeneity/uniformity as well as artifact characteristics^13,16^. These can be readily measured and thus image quality objectively determined. One of the fundamental principles for radiographic imaging is optimization, meaning that the imaging is to be performed using doses that are as low as reasonably achievable (ALARA), consistent with the diagnostic task. Additional roentgen radiation for a conventional two-dimensional periapical radiograph may be avoided when a CBCT of the patient already exists; the potential for determining the root canal length has been confirmed. When the three-dimensional area is understood, the accuracy of endodontic working length measurement can be increased^7, 8^. Hence, in the context of optimization it would be very helpful to establish a link between image quality parameters (and thus exposure parameters as well) and the requirements of a specific diagnostic task. The question that arises is how much the measurement of the working length is influenced by the practitioner as well as the CBCT device type. Therefore, the aim of this study was to investigate potential correlations between objective CBCT image parameters and accuracy in endodontic working length determination using a sufficiently large number of experienced observers.

## Materials and methods

### Image quality parameters

The noise, contrast and spatial resolution as objective image parameters were investigated. To ensure realistic image quality, a simulated attenuation of the human head tissues by means of a 16 cm diameter plastic water-filled tank into which each of the phantoms was submerged for the CBCT scan. Noise was defined by means of a low-contrast phantom (Ø 80 mm) made of polymethylmetarcylate (PMMA) and containing an insert (Ø 10 mm) filled with air. This was related to contrast as the well-established contrast-noise-ratio (CNR): 1. Thus, the mean expresses the mean grey value in the CBCT image. The value for water was determined by means of the water-filled tank (Ø 16 cm). A higher value expresses higher contrast in relation to noise, i.e. less noise in relation to image content.

Spatial resolution was assessed as approximated Modulation Transfer Function (MTF)^17^ as derived from a slanted metal-edge image. The phantom for this purpose was a sharp lead edge (8 cm length) mounted onto a polymethylmethacrylate-plate of 10 mm thickness. Although the concept of MTF is complex, the output of MTF measurements essentially represents the (standardized) spatial resolution in relation to signal modulation (≈ contrast). It is the standard spatial resolution measure in digital radiography and photography today and is notated in cycles/mm^18^. Its output can be safely translated into the well-known measure "linepairs/mm" [Lp/mm]. We used this measure at 10% modulation (≈ contrast) as the figure of merit for our evaluation.

### CBCT machines

A total of seven different CBCT machines; 3D Accuitomo 80 (J Morita Corp, Kyoto, Japan), 3D eXam (KaVo Dental, Biberach, Germany), Veraviewepocs 3D R100 (J Morita Corp, Kyoto, Japan), PaX-Duo3D (Vatech, Gyeonggi-do, Korea), Scanora 3Dx (Sorodex, Tuusula, Finland), ProMax 3D Mid (Planmeca Oy, Helsinki, Finland) and Orthophos SL (Dentsply Sirona, Bensheim, Germany). The settings are listed in Table 1. The data were exported as DICOM-files.

**Table 1:** CBCT devices and parameters used for the evaluation. FOV: field of view. Voxel size provided as stated by the manufacturer.

CBCT device	kV	mA	Scan-time (s)	FOV (mm)	Voxel size (mm)
3D Accuitomo 80 (J Morita Corp, Kyoto, Japan)	90	8	17.5	80 × 80	0.160
3D eXam (KaVo Dental, Biberach, Germany)	120	5	14.7	160 × 80	0.20
Veraviewepocs 3D R100 (J Morita Corp, Kyoto, Japan)	90	8	9.4	100 × 80	0.125
PaX-Duo3D (Vatech, Gyeonggi-do, Korea)	90	8	24	85 × 85	0.2
Sanora 3Dx (Sorodex, Tuusula, Finland)	90	8	20	100 × 80	0.15
ProMax 3D Mid (Planmeca Oy, Helsinki, Finland)	90	8	12	80 × 80	0.14
Orthophos SL (Dentsply Sirona, Bensheim, Germany)	85	7	14.2	80 × 80	0.160

### Tooth phantom

A total of 10 single-rooted mandibular human teeth (2 left and right premolars, canines and front teeth ea.) collected from an oral surgery department of a German university dental school for reasons (usually periodontal, endodontic, orthodontic and traumatic) unrelated to this investigation and stored in 4% formaldehyde solution until use. This research material was considered, according with the corresponding authorities’ legal regulations, as so-called excess material. An endodontic access cavity was prepared with a diamond bur. The pulp tissue was removed and the root canal patency probed to the physiological foramen with a K-file, ISO 15 (VDW, Munich, Germany). The actual working length was determined by placing a K-type file in the root canal and under microscopic observation (16x; Stemi DRC; Carl Zeiss Jena, Jena, Germany) until its tip reached the physiological foramina limit^3^. As radiological reference for the assessment of the actual working length at the physiological foramen, a small steel sphere (Ø 0.5 mm) was adhesively fixed in the access cavity. The apical end-point was defined as the physiological foramen^3^.

**Figure 1:**
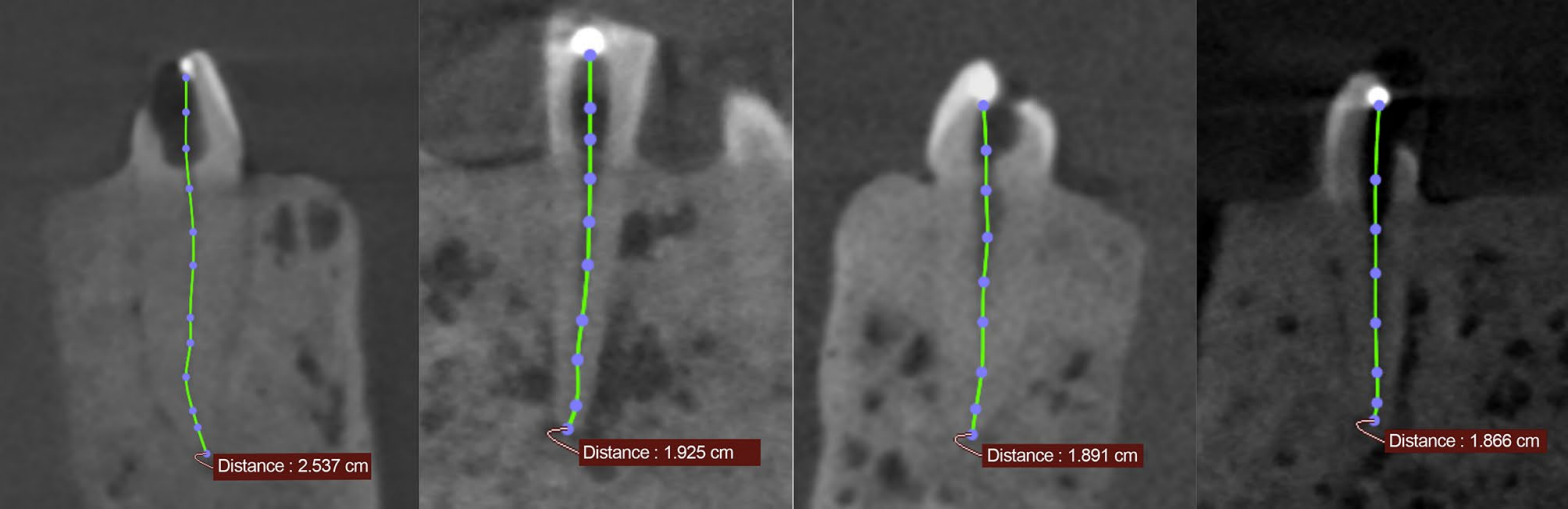
Resulting (exemplary) CBCT scans with the polygon measurement tool.

To simulate the bony tissue, a sponge was cut into the shape of an approximate mandible and small incisions were made at the respective tooth positions. After placing the teeth roughly at their natural positions, the phantom was then fixed in dental stone (100 g plaster added to 100 ml water; Moldasynt, Heraeus Kulzer, Hanau, Germany). After hardening, the surface of the phantom was coated with a thin (< 1 mm) layer of dental wax simulating the soft tissue and also isolating the model from water. The reasoning behind this model was to obtain a radiographically relatively realistic model with well-adapted teeth in a bone-mimicking support. The procedure resulted in one model containing a total of 10 mandibular teeth (four incisors, two canines and four premolars [Fig. 1]).

### Working length assessment

The true length of the K-files (31 mm) used for working determination was assessed by advancing the file from the coronal reference point (steel sphere) to the physiological foramen through control by a digital microscope (10x; VHX-1000D, Keyence, Osaka, Japan). The file was marked and subsequently cut at this point; thus, the resulting working length measured with a micrometer.

Within the CBCT data, the DICOM-files were imported into OsiriX (Version 7.0.2; Pixmeo SARL, Bernex, Switzerland). Multiplanar reconstructions (MPR) were used as the basis for the evaluation. The data were reoriented until the coronal and the apical point of reference of the respective tooth were visible in all three planes. Observers were allowed to adapt/modify windowing and levelling of the grey values on the screen. By means of the "Opened Polygon" tool, the observers were instructed to place points with the mouse-driven cursor in order to delineate the curve of the root canal as accurately as possible. The number of points placed was not limited, and every reference point besides the coronal and apical ones needed to be pointed out in all cases. The software then provided a computed length based on the polygon formed by the points between apical and coronal reference points. Error was defined as the difference between true working length and CBCT-based measured working length.

### Sample size

Sample size might be calculated taking into account the following factors, such as time limitation of the observers to avoid fatigue (30 min)^19^ and the number of observers (seven). The sample size calculation was performed using OpenEpi (Version 3.01, open source epidemiologic statistics for public health, last access 10th September 2020: https://www.openepi.com/Menu/OE_Menu.htm) open source software, using the 70 teeth enrolled as the population and then 60% of the hypothesized frequency. Fatigue is a determinant factor in radiographic diagnosis, often referred to as sensitivity or vigilance decrease^19^. The sample size of confidence level of 99.99% was set at 67 teeth; the authors decided to enroll all teeth into the investigation. Thus, the approximate total evaluation time when all 70 samples are observed should be about 35 min per observer.

### Observers/observations

A total of seven observers with at least five years of working experience and with CBCT working experience were selected for this investigation. All observers were trained on the OsiriX software (Version 7.0.2; Pixmeo SARL, Bernex, Switzerland) and calibrated by having them assess one CBCT case that was not entered into the evaluation. The order of the CBCTs was randomized for each observer and observation. To assess intra-operator-reproducibility, the procedure was repeated within six weeks again with a randomized order of CBCT images.

### Statistical evaluation

The endpoint variable was accuracy, i.e. the deviation of the CBCT-measured K-file length from the actual working length. Using R language and environment for statistical computing^20^, errors were descriptively evaluated across all observers and observations. By means of multiple (MANOVA) analysis of variance, errors were compared between observers, different CBCT devices and objective image quality parameters (CNR, MTF). Spearman correlation was used to evaluate potential interactions. The level for statistical significance was set to 0.05. Intra-operator-reproducibility was assessed by means of intraclass correlation (ICC). The 95%-confidence interval (95%-CI) of the ICC was also computed. The operators were treated as randomly selected from a larger population of operators with similar characteristics, thus suggesting a two-way random effects model^21,22^.

## Results

**Figure 2:**
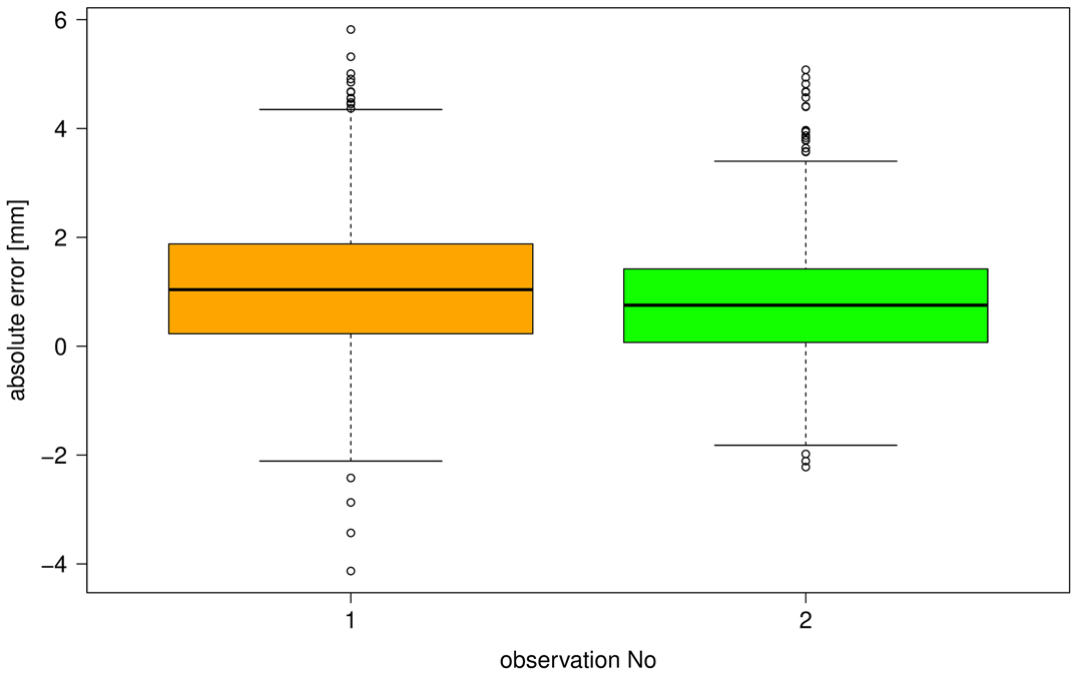
Box plots of the absolute errors pooled over the seven observers for the first and second observation. Boxes represent the data between the 25% and the 75%-quartiles, while whiskers represent the highest and lowest values except for outliers. The latter are those values lying outside 1.5 times the interquartile range above the upper quartile and below the lower quartile.

**Figure 3:**
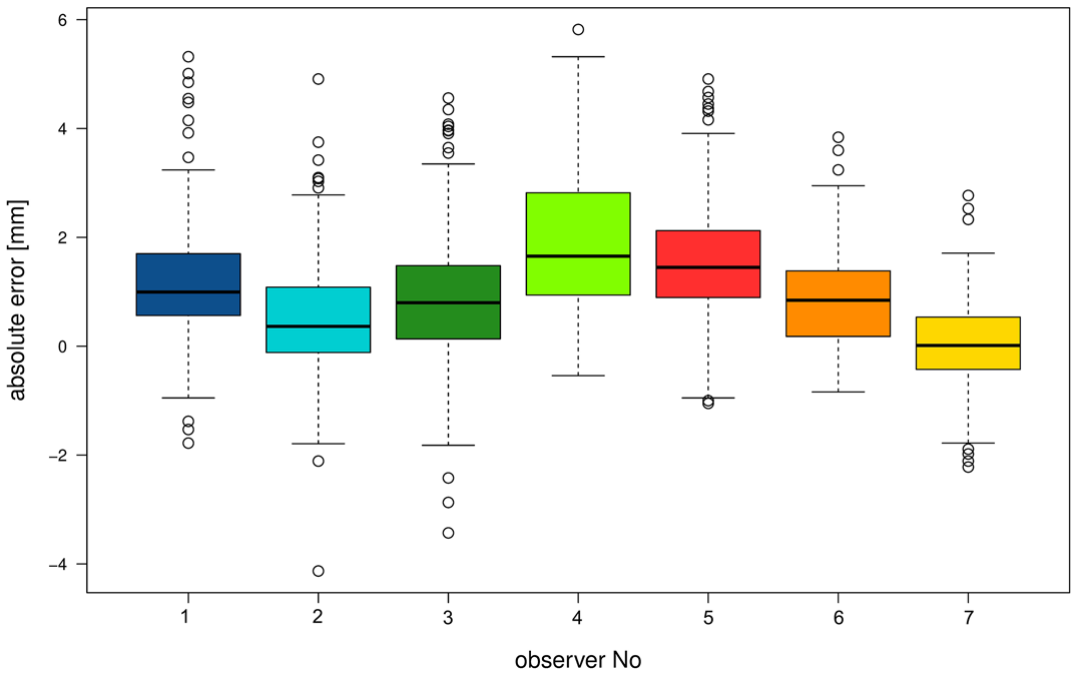
Box plots of the absolute errors separated for the seven observers, yet pooled over the two observations. Boxes represent the data between the 25% and the 75%-quartiles, while whiskers represent the highest and lowest values except for outliers. The latter are those values lying outside 1.5 times the interquartile range above the upper quartile and below the lower quartile.

CBCT devices and parameters used for the evaluation are shown in Table 1; the voxel size provided is stated by the corresponding manufacturer. Objective image quality parameters are displayed in Table 2. CNR ranged from 0.18 (PaX-Duo3D) to 1.94 (3D eXam), while MTF 10% ranged from 1.8 cycles/mm (3D eXam) to 2.8 cycles/mm (Orthophos SL). Absolute errors pooled over all devices, observers and both observations ranged between − 4.13 mm to 5.82 mm (mean: 1.16 mm, median: 1.04 mm). This corresponds to a relative error ranging between − 21.1% and 24.5% (mean: 4.4%, median: 4.1%). Errors varied significantly between both observations (*p* < 0.001, Fig. 2) and observers (*p* < 0.001, Fig. 3), yet not between CBCT devices. Multivariate analysis (MANOVA) did not reveal an influence of the combined image quality factors CNR and MTF on measurement accuracy. Inter-operator reproducibility as assessed between the two observations was poor, with a mean ICC of 0.48 (95%-CI 0.38, 0.59) in the first observation and 0.40 (95%-CI 0.30, 0.51) in the second observation. Intra-operator reproducibility pooled over all observers as compared between both observations was only moderate with a mean ICC of 0.58 (95%-CI 0.52 to 0.64).

**Table 2:** Objective image quality parameters. CNR: contrast to noise ratio (Eq. 1), MTF 10%: approximated modulation transfer function at 10% modulation.

CBCT device	CNR (air–water)	MTF 10% (Lp/mm)
3D Accuitomo 80 (J Morita Corp, Kyoto, Japan)	0.67	2.4
3D eXam (KaVo Dental, Biberach, Germany)	1.94	1.8
Veraviewepocs 3D R100 (J Morita Corp, Kyoto, Japan)	1.03	2.1
PaX-Duo3D (Vatech, Gyeonggi-do, Korea)	0.18	2.3
Scanora 3Dx (Sorodex, Tuusula, Finland)	0.91	2.3
ProMax 3D Mid (Planmeca Oy, Helsinki, Finland)	0.85	2.5
Orthophos SL (Dentsply Sirona, Bensheim, Germany)	0.79	2.8

## Discussion

CBCT has become established as a 3D radiographic technique in endodontic treatment^9^ and is described as a helpful, accurate and reliable method^7,8,10,23^. According to the AAE/AAOMR joint position statement, it should be used "only when the need for imaging cannot be met by lower dose two-dimensional (2D) radiography"^24^. A recent review concluded that "diagnostic CBCT may provide additional information when compared to PR, which may have an impact on the treatment planning of complex endodontic re-treatment cases"^25^. Obviously, a clinician will expect high-quality images when deriving treatment plans or diagnosis from them. From a technical perspective, CBCT image quality can be determined using objective image quality parameters such as noise, contrast and spatial resolution^14,15^. Since MTF relates spatial resolution to contrast and CNR provides a good estimate of the noise level, the image quality parameters used for this study should sufficiently summarize the objective quality of the CBCT images. However, a common problem in clinical radiology is the missing link between objective image quality parameters and a specific diagnostic task^26^. One key principle in radiation protection refers to optimization of the exposure in the sense of the well-known "as low as reasonably achievable-(ALARA)" principle^27^. Thus, knowing which quality is sufficient for a specific diagnostic or treatment task is essential for adopting this radiation protection principle. This investigation was aimed to relate objective image quality to the relatively simple-appearing task of endodontic root-canal length assessment. The endpoint variable (length) is interval-scaled and thus very suitable for statistical analysis. Interestingly, we found no dependency between accuracy and either of the two objective quality measures despite the fact that at least the CNR differed vastly between the CBCTs. This is interesting, as exposure parameters (e.g. kilo voltage, milliampere, voxel sizes, Table 2) as well differed significantly. Instead, a significant dependency between accuracy and observer in combination with poor inter-operator reproducibility was observed. This is a common finding in radiographic image interpretation^28,29^. This observation is also apparent in endodontic radiography^30^. The results of this research indicate, however, that the task to determine a length of a curved K-file in a 3D-image is beset with additional inherent difficulties. This assumption is also supported by the wide error range, from an overestimation of true length (4.13 mm) to a significant underestimation (5.82 mm). Mean and median indicate a slight underestimation of approximately 1 mm. As only single-rooted teeth by a total of seven experienced observers were assessed in the present study, these errors appear substantial. Connert et al.^9^, based on only two observers, reported similar results. The reasons for such substantial differences could be explained through the factors discussed above and that seven observers will likely differ in their radiographic interpretation. Furthermore, studies with similar results evaluating single-rooted teeth were performed by a single observer^7,8^. Connert et al.^9^ used the Euclidean distance between two end-points for their results assessment, while the observers in this investigation were asked to follow the curved line of the canal by placing intermediate points. This procedure will likely lengthen the measured distance, which explains overestimation. It may well be speculated that the working length assessment method in this investigation, despite aiming to provide more accurate results, instead introduced an additional error. A research design comparing the method used in this investigation and where only two or three points are used to define the root canal curve seems to be advisable in order to further support this assumption. The significant inter-observer error, however, once again highlights the well-known fact that a sufficient number of observers is fundamental for radiographic image assessment^31^. However, from a clinical point of view, it is important to stress out that since nowadays patients often bring an already existing CBCT of the region of interest with them, thus, the clinician should always be aware that the image quality variability is highly depending not only on the type of machine used but also on the settings used at the time of generating the mages.

In vivo, the influence of inevitable slight patient motion will introduce additional motion blur in the reconstruction and thus deteriorate image quality by reducing spatial resolution^32^. Small voxel sizes increase this effect^16^. Whether novel approaches to correct for such errors^33^ are effective remains unknown so far. It can be assumed that the task of accurately measuring an endodontic working length by means of CBCT data is more challenging in vivo than in our ex vivo setup. This should be born in mind when such a radiographic task is required in a clinical scenario. However, it must be noted that a precise differentiation between major and minor foramen in the apical region is not possible with CBCT, due to that root canal morphology has a statistically significant influence on the measurement^34^. The described similar precision working length determination with CBCT and electronic apex locators reported by Jeger et al.^8^ cannot be confirmed by this investigation. Although the actual working length in this investigation was determined taking the physiological foramina as reference point and under magnification, it would certainly be interesting to design a research model in which it would be established up to what point a CBCT working length determination could substitute or enhance an apex locator established one. The mean distances between the physiological foramen (apical constriction) and the anatomical apex (major foramen) can vary from 0.43 mm to 1.02mm^3^. In the investigated CBCT devices, the difference between working length and physiological foramen (apical constriction) could neither be measured nor distinguished due to the lack of imaging accuracy. Therefore, with CBCT only the radiological discernible root canal length could be evaluated and not on the working length was focused in this research. Although a high intra-operator reliability for CBCT scans was observed unaffected by patient gender and age as well as the number of root canal curvatures, future studies with larger sample sizes are recommended. As to the results obtained in this research, it must be questioned and further investigated whether existing CBCT scans could be used for endodontic working length determination in multi-rooted teeth or would even have the potential to replace conventional periapical diagnose radiographs for this purpose^8^.

## Conclusions

Within the limitations of the study, it can be concluded that objective image quality and exposure parameters seem not to have a significant influence on accuracy in determining endodontic working lengths in CBCT scans. The main factor of variance is the observer, which supports the well-established concept that studies require a sufficient number of observers to produce reliable and accurate results.

## Data Availability

The datasets generated during and/or analyzed during the current study are available from the corresponding author on reasonable request.
